# Identification of QTL and Candidate Genes Controlling Plant Height and Internode Length in a Newly Characterized Bread Wheat Recombinant Inbred Population

**DOI:** 10.3390/genes17050567

**Published:** 2026-05-17

**Authors:** Zidong Wan, Shuai Ge, Mengxin Li, Xinyan Wang, Dongjie Cui, Qing Chi, Bing Li, Hangbo Xu, Jialing Lu, Zhen Jiao, Wenhui Wei, Panfeng Guan

**Affiliations:** 1 Henan Key Laboratory of Ion-beam Green Agriculture Bioengineering, School of Agriculture and Biomanufacturing, Zhengzhou University, Zhengzhou 450001, China; wanzidong001@gmail.com (Z.W.); welcome1019@163.com (S.G.); mengxinli7@163.com (M.L.); 15737335257@163.com (X.W.); cuidongjie0323@zzu.edu.cn (D.C.); qchi@zzu.edu.cn (Q.C.); 006805@yzu.edu.cn (B.L.); xuhangbo@126.com (H.X.); lujialing92@zzu.edu.cn (J.L.); jiaozhen@zzu.edu.cn (Z.J.); 2 College of Agronomy and Life Sciences, Zhaotong University, Zhaotong 657000, China

**Keywords:** bread wheat, plant height, internode length, QTL, candidate genes, liquid-phase chip

## Abstract

**Background:** Internode length (IL), a key component of plant height (PH), plays an important role in achieving the optimal architecture in wheat. However, the genetic mechanisms underlying internode elongation are not well understood. **Methods:** In this study, a recombinant inbred line (RIL) population derived from a cross between Bainong 4199 (BN4199) and Zhengyinmai 2 (ZYM2) was evaluated for PH and five ILs across two field locations over two years and genotyped using a 120 K liquid-phase chip. **Results:** A total of 141 quantitative trait loci (QTL) associated with PH and the five ILs were mapped onto 20 chromosomes, except for chromosome 5D. Among these, 37 stable QTL were identified on chromosomes 1B, 2B, 2D, 4B, 5A, 7A, 7B and 7D, accounting for 3.86–25.97% of the phenotypic variation. Meanwhile, 23 co-localized QTL associated with at least two traits were detected, with QTL cluster regions on chromosomes 2D, 4B, 5A, 7A, and 7B. Moreover, the total additive effects of the QTL combinations increased with the number of QTL, which indicates the effectiveness of pyramid breeding. Additionally, based on gene function annotation, the cloning and characterization of rice orthologs, and analysis via the QTG miner module of the wheat integrative gene regulatory network (wGRN) platform, 63 candidate genes (e.g., *Rht1*, *Rht8*, *TB1* and *ZnF-B*) were prioritized within the stable QTL intervals, and their tissue expression patterns were analyzed. **Conclusions:** Collectively, these findings not only deepen our understanding of the genetic basis of PH and ILs in wheat but also lay a foundation for the further validation and functional characterization of candidate genes, enabling the optimization of plant architecture through marker-assisted selection (MAS) to ultimately improve agronomic performance and yield potential.

## 1. Introduction

Bread wheat (*Triticum aestivum* L.) is one of the world’s most important cereal crops, and increasing its yield is crucial for addressing the challenges of global population growth and the food crisis [1]. During the first ‘Green Revolution’, dwarf breeding established the semi-dwarf ideotype of wheat, dramatically increasing wheat yield and harvest index [2,3,4,5]. The ideotype can improve light energy conversion efficiency, reduce pest and disease incidence, and enhance seed yield in crops [6]. Plant height (PH) is a key trait in ideotype improvement in wheat [6,7]. However, the widely used ‘Green Revolution’ genes, *Rht1* (*Rht-B1b*) and *Rht2* (*Rht-D1b*), not only reduce plant height but also decrease coleoptile length, grain weight, and nitrogen-use efficiency [8,9,10]. Therefore, the discovery and utilization of new dwarfing loci and genes have long been a research hotspot in wheat breeding.

Wheat plant height is morphologically divided into two components: spike length (SL) and the total length of the elongated internodes above ground [11,12]. The desirable PH in wheat breeding programs is, therefore, achieved by optimizing these components. Previous studies have shown that cultivars with shorter basal internodes are more likely to exhibit lodging resistance [13,14]. Classical genetic studies have demonstrated that PH is a complex quantitative trait influenced by both Mendelian genes and quantitative trait loci (QTL) [12,15]. Up until now, 28 wheat dwarfing genes have been formally catalogued [16,17]. Among them, the cloned and validated genes are *Rht1*, *Rht2*, *Rht3* (*Rht-B1c*), *Rht8*, *Rht10* (*Rht-D1c*), *Rht11* (*Rht-B1e*), *Rht12*, *Rht13*, *Rht17* (*Rht-B1p*), *Rht18*/*Rht24*, *Rht23* (*5Dq*) and *Rht25* [18,19,20,21,22,23,24,25,26,27,28,29,30]. Furthermore, a number of QTL associated with PH have been identified across 21 wheat chromosomes through linkage mapping in biparental populations and association mapping in natural populations [31]. In particular, multivariate conditional QTL frameworks have proven valuable for partitioning PH into spike and internode contributions, thereby separating loci with direct effects on stem elongation from those acting indirectly through correlated components [32]. In addition, increased activity of the *TEOSINTE BRANCHED1* (*TB1*) gene has been shown to restrict stem height and elongation in bread wheat [11].

Genetic variations that cause semi-dwarfism in wheat, barley and rice primarily affect the biosynthesis, metabolism or signal transduction of the plant hormone gibberellin (GA) and brassinosteroids (BRs) [31,32]. The tall alleles *Rht-B1a*/*Rht-D1a* encode normal DELLA repressors that are degraded after forming the GA–GID1–DELLA complex, thereby releasing GA-promoted growth [26,28]. By contrast, major “Green Revolution” and related loci—*Rht1*, *Rht2*, *Rht3*, *Rht11*, and *Rht17*—produce DELLA variants that cannot be degraded and, thus, constitutively repress GA responses, reducing stem elongation [18,22]; *Rht10* (*Rht-D1c*) further amplifies this effect via tandem duplication of the *Rht-D1b* region. Other dwarfing mechanisms include reduced bioactive GA via GA2ox genes (*Rht12*: *GA2ox-A13*; *Rht18*/*Rht24*: *GA2ox-A9*), GA-pathway modulation by *Rht8*, altered miR172–AP2 regulation at *Rht23* (5Dq), and restricted cell expansion through immune/cell-wall reinforcement at *Rht13* [19,20,21,23,27,29,33]. In addition, the *Rht25* gene, which encodes a plant-specific AT-rich sequence- and zinc-binding protein (PLATZ), reduces plant height by interacting with DELLA [30], while the GSK3/SHAGGY-like kinase can phosphorylate and stabilize Rht-B1b protein, thereby strengthening dwarfism [34]. Notably, a 4BS semi-dwarf haplotype involving Rht-B1b/EamA-B/ZnF-B highlights GA–BR crosstalk, where ZnF positively regulates BR signaling by directly interacting with TaBRI1 and TaBKI1 [9,35]. Furthermore, natural deletion of the ‘r-e-z’ haploblock not only results in a semi-dwarf phenotype but also increases wheat yield by 6.48–15.25% and improves the low nitrogen (N)-use efficiency (NUE), which establishes a molecular framework for engineering BR-driven cereal ideotypes [9].

In this study, variations in PH and its component traits, internode length (IL), were evaluated in a recombinant inbred line (RIL) population derived from the cross between Bainong 4199 (BN4199) and Zhengyinmai 2 (ZYM2) across multiple field environments. Furthermore, a high-density linkage map was constructed using a 120 K liquid-phase chip to genotype the BN4199/ZYM2-RIL population. The objectives of this study were to detect genetic loci associated with PH and IL and to identify putative candidate genes within QTL regions. These findings will deepen our understanding of the genetic basis of wheat plant height development and facilitate the optimization of plant architecture for developing new varieties with high and stable yields.

## 2. Materials and Methods

### 2.1. Plant Materials

The wheat RIL population used in this study consisted of 184 lines derived from a cross between BN4199 and ZYM2. The female parent, BN4199, was developed by the Wheat Research Center of Henan Institute of Science and Technology. It is a high-light-efficient wheat variety widely cultivated in the North China Plain. The male parent, ZYM2, is an exotic wheat line characterized by a tall stem and a high grain number per spike.

### 2.2. Field Trials and Phenotyping

During the 2023–2024 and 2024–2025 growing seasons, the BN4199/ZYM2-RIL population, along with the two parents, was planted at the Xingyang (XY) and Xuchang (XC) experimental sites in Henan Province, China. These sites are hereafter referred to as E1 (XY24), E2 (XC24), E3 (XY25) and E4 (XC25). The field trials were conducted in a randomized complete block design with three repetitions. Each row is 1.5 m long, with a row spacing of 0.3 m, and each row contains 30 seeds. Crop management, including irrigation, pesticide and fertilizer applications, followed local standard cultivation practices. At maturity, PH and IL were measured for six to ten randomly selected plants per each RIL in each repetition. Plant height was measured from the ground to the tip of the spike, excluding awns. The first internode length (IL1) was measured from the base of the spike to the first node from the top. The second internode length (IL2) was measured from the first node to the second node, and similarly for the third (IL3), fourth (IL4) and fifth (IL5) internode lengths.

### 2.3. Genotyping and Linkage Map Construction

Genomic DNA was extracted from fresh seedling leaves of the RILs and both parents using the CTAB method [36]. The wheat 120 K liquid-phase (120 K–4 HWA) chip from the Department of Life Science, Tcuni Inc., Chengdu, China (https://www.tcuni.com), was used for genotyping. Redundant markers with missing data (>10%) or distorted segregation (*p* < 0.001) were filtered using the ‘BIN’ function in QTL IciMapping v4.2 software [37]. A genetic linkage map was then constructed using the ‘MAP’ function. The graphical presentation of the linkage map was generated using Mapchart v.2.3 software [38]. The physical positions of the molecular markers were based on the International Wheat Genome Sequencing Consortium (IWGSC) RefSeq v2.1 genome of Chinese Spring (CS) hexaploid wheat.

### 2.4. QTL Analysis

QTL analysis was performed using Windows QTL Cartographer version 2.5 [39]. Composite interval mapping (CIM) method with logarithm of odds (LOD) score threshold of 2.5 was used to detect significant QTL. A forward and backward regression model was applied with a window size of 10 cM, a walking speed of 2 cM, five control markers, 1000 permutations, and a significance level of 0.05. QTL detected in two or more environments were considered stable QTL in this study. QTL that were less than 1 cM apart or shared common flanking markers were regarded as a single locus and named according to the International Rules of Genetic Nomenclature adapted for wheat [40,41].

### 2.5. Identification of Candidate Genes and RT-qPCR Analysis

High-confidence genes within the stable QTL regions were obtained through the WheatOmics 1.0 platform (http://wheatomics.sdau.edu.cn/) based on the annotation of the IWGSC RefSeq v2.1 genome [42]. The ‘Triticeae-GeneTribe’ database (https://wheat.cau.edu.cn/TGT/) was used for homologous gene query and gene ontology (GO) annotation [43]. The China National Rice Data Center (https://www.ricedata.cn/) was employed to retrieve information on the cloning and functional annotation of homologous genes in rice. The potential candidate genes were narrowed down based on their functional annotation, protein family classification, and predicted regulatory roles. Furthermore, candidate genes within the QTL intervals were prioritized using the wheat integrative gene regulatory network (wGRN) platform (http://wheat.cau.edu.cn/wGRN) via the QTG miner module [44]. The tissue- and stage-specific expression data of candidate genes were obtained from the WheatOmics 1.0 platform (http://wheatomics.sdau.edu.cn/) [42]. The reverse transcription–quantitative polymerase chain reaction (RT-qPCR) analysis of candidate genes was performed using the parental stem at the jointing stage.

### 2.6. Statistical Analysis

Analysis of variance (ANOVA), best linear unbiased estimation (BLUE), and broad-sense heritability (*H*^2^) were performed using the ‘AOV’ function in the QTL IciMapping v4.2 software [37]. The Pearson correlation analysis was conducted using the ‘PerformanceAnalytics’ R package (https://CRAN.R-project.org/package=PerformanceAnalytics). The Student’s *t*-test (*p* < 0.05) was performed on the phenotype values using IBM SPSS Statistics v22.0 (SPSS Inc., Chicago, IL, USA). Origin 2018 software (OriginLab, Northampton, MA, USA) was used to generate the figures.

## 3. Results

### 3.1. Phenotypic Evaluation of RIL Population

**Figure 1:**
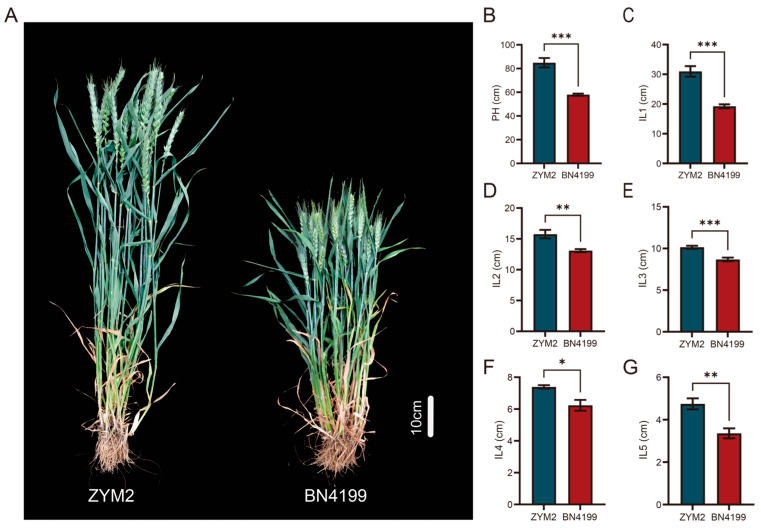
Phenotype comparison of plant height (PH, (**A**,**B**)) and five internode lengths (IL1–IL5, (**C**–**G**)) between the parents ZYM2 and BN4199. *, **, ***: significant differences at the *p* < 0.05, *p* < 0.01 and *p* < 0.001 levels, respectively.

**Figure 2:**
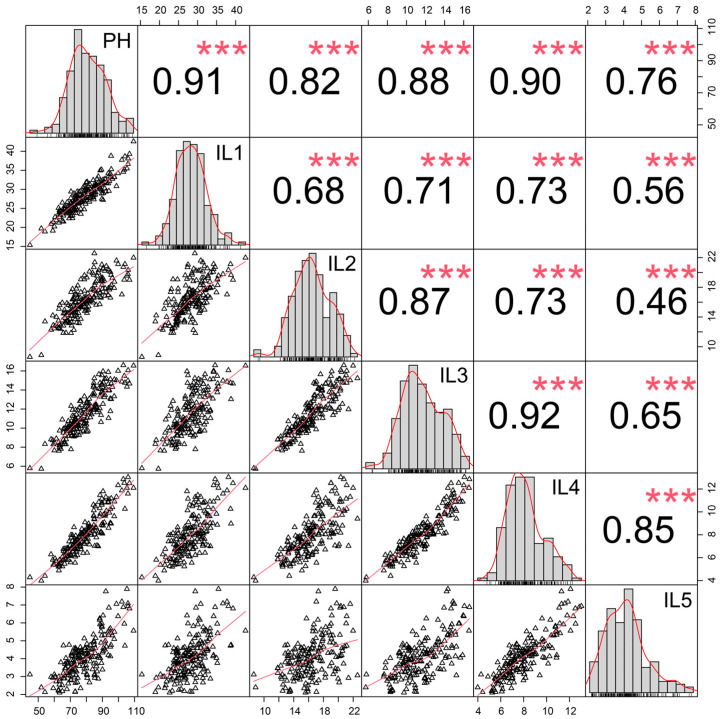
Correlation analysis of plant height-related traits in the ZYM2/BN4199-RIL population using BLUE values. ***: Significant at the *p* < 0.001 level.

The PH and the five ILs of the BN4199/ZYM2-RIL population were evaluated across four different field environments (Supplementary Table S1). Compared to BN4199, ZYM2 had longer internodes, resulting in a greater PH (Figure 1). Transgressive segregation for PH and the five ILs was observed in the RIL population across all environments, with all traits showing considerable variation. Furthermore, the BN4199/ZYM2-RIL population exhibited continuous variation and a nearly normal distribution for PH and the five ILs, consistent with the quantitative trait characteristics and suitable for subsequent QTL mapping (Table S1, Figure 2). The ANOVA of PH and its component traits in the RIL population under different environmental conditions showed that PH and the five ILs were significantly affected by genotype, environment, and their interaction (Table 1). Meanwhile, the *H*^2^ of PH and the five ILs in the BN4199/ZYM2-RIL population all exceeded 80%, indicating high genetic stability (Table 1). In addition, significant and positive correlations (*p* < 0.001) were observed among PH and the five ILs’ traits based on BLUE values, suggesting that these traits are interrelated (Figure 2).

### 3.2. QTL Mapping for PH and IL Traits

**Figure 3:**
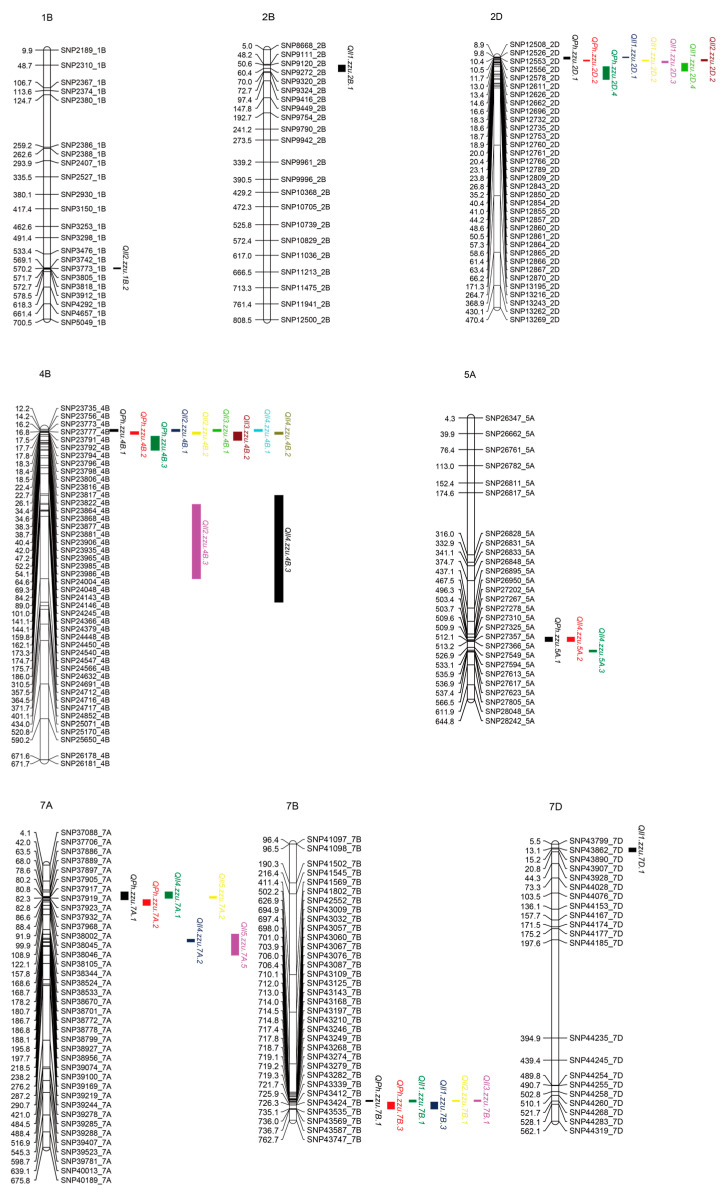
Distribution of stable QTL on chromosomes.

Based on the phenotypic data and BLUE values across all field environments, a total of 141 QTL associated with PH and the five ILs were detected in the BN4199/ZYM2-RIL population across 20 chromosomes, with the exception of chromosome 5D (Supplementary Table S2). The D sub-genome had fewer QTL (41) than the A (49) and B (51) sub-genomes, while the greatest number of QTL (21) were distributed on chromosomes 2D and 7A. The phenotypic variance (PVE) explained by each individual QTL ranged from 3.60% to 25.97%. In this study, QTL detected in two or more environments were considered stable (Table 2, Figure 3), and QTL located within the same interval were referred to as co-localized QTL (Table 3).

For PH, a total of 26 QTL were identified on chromosomes 1D, 2D, 3D, 4A, 4B, 4D, 5A, 7A, and 7B, among which eleven stable QTL were located on chromosomes 2D, 4B, 5A, 7A and 7B. One of these stable QTL, namely, *QPh.zzu.2D.1*, was detected in all environments and in the BLUE analysis, with LOD scores ranging from 4.60 to 7.39 explaining 7.93% to 12.95% of the PVE. The favorable alleles that reduce PH for the stable QTL *QPh.zzu.2D.1*, *QPh.zzu.2D.2*, *QPh.zzu.2D.4*, and *QPh.zzu.5A.1* were contributed by BN4199, whereas ZYM2 contributed the favorable alleles for *QPh.zzu.4B.1*, *QPh.zzu.4B.2*, *QPh.zzu.4B.3*, *QPh.zzu.7A.1, QPh.zzu.7A.2, QPh.zzu.7B.1* and *QPh.zzu.7B.3*.

For IL1, a total of 24 QTL were identified on chromosomes 1D, 2B, 2D, 3D, 4B, 5B, 6A, 6B, 7A, 7B, and 7D. Among them, the stable QTL *QIl1.zzu.2B.1, QIl1.zzu.2D.1*, *QIl1.zzu.2D.2*, *QIl1.zzu.2D.3*, *QIl1.zzu.2D.4* and *QIl1.zzu.7D.1* carried favorable alleles from BN4199, whereas the favorable allele for the stable QTL *QIl1.zzu.7B.1* and *QIl1.zzu.7B.3* came from ZYM2. Notably, *QIl1.zzu.2D.1* and *QIl1.zzu.2D.2* were consistently identified across two different environments and in the BLUE analysis, with *QIl1.zzu.2D.2* exhibiting the highest PVE of up to 25.97%.

For IL2, a total of 22 QTL were identified on chromosomes 1A, 1B, 1D, 2D, 3A, 3B, 3D, 4A, 4B, 5A, 6A, 6D and 7B. Six of these QTL were stable and mapped on chromosomes 1B, 2D, 4B and 7B. The increasing allele of *QIl2.zzu.2D.2* was contributed by ZYM2, whereas BN4199 contributed the increasing alleles at the remaining five loci. Among these, *QIl2.zzu.7B.1* exhibited the strongest signal, with LOD values ranging from 2.79 to 6.38 and explaining 5.27% to 11.33% of the phenotypic variance.

For IL3, a total of 21 QTL were identified on chromosomes 1A, 1B, 1D, 2A, 4A, 4B, 4D, 5A, 5B, 6A, 6B, 6D, 7A and 7B. Three stable QTL were detected on chromosomes 4B and 7B, namely, *QIl3.zzu.4B.1*, *QIl3.zzu.4B.2* and *QIl3.zzu.7B.1*. The increasing alleles at all three loci were contributed by BN4199. Notably, *QIl3.zzu.4B.1* was identified as the most robust locus, being consistently detected across all environments and in the BLUE analysis, with LOD values ranging from 3.38 to 7.62 and accounting for 6.14% to 14.17% of the phenotypic variance.

For IL4, a total of 24 QTL were identified on chromosomes 1A, 1B, 2D, 3B, 4A, 4B, 4D, 5A, 6B, 6D, 7A and 7B. Seven of these QTL were stable and located on chromosomes 4B, 5A and 7A. The increasing alleles at *QIl4.zzu.5A.2*, *QIl4.zzu.5A.3* and *QIl4.zzu.7A.2* were contributed by ZYM2. By contrast, increasing alleles at *QIl4.zzu.4B.1*, *QIl4.zzu.4B.2*, *QIl4.zzu.4B.3* and *QIl4.zzu.7A.1* were contributed by BN4199. Among these loci, *QIl4.zzu.7A.1* displayed the largest effect, with LOD values ranging from 5.14 to 7.31 explaining 9.24% to 13.09% of the phenotypic variance.

For IL5, two stable QTL were identified on chromosome 7A, namely, *QIl5.zzu.7A.3* and *QIl5.zzu.7A.6*. The major locus, *QIl5.zzu.7A.3*, was detected in E1, E2, E4 and the BLUE analysis, with LOD values ranging from 6.23 to 11.90 and accounting for 11.09% to 20.33% of the phenotypic variance. The increasing allele was contributed by BN4199. The *QIl5.zzu.7A.6* was detected in E2, E4 and BLUE, with LOD values ranging from 3.30 to 6.25 and explaining 7.14% to 11.11% of the phenotypic variance. The increasing allele was contributed by ZYM2.

### 3.3. Additive Effects Analysis of the Stable QTL

**Figure 4:**
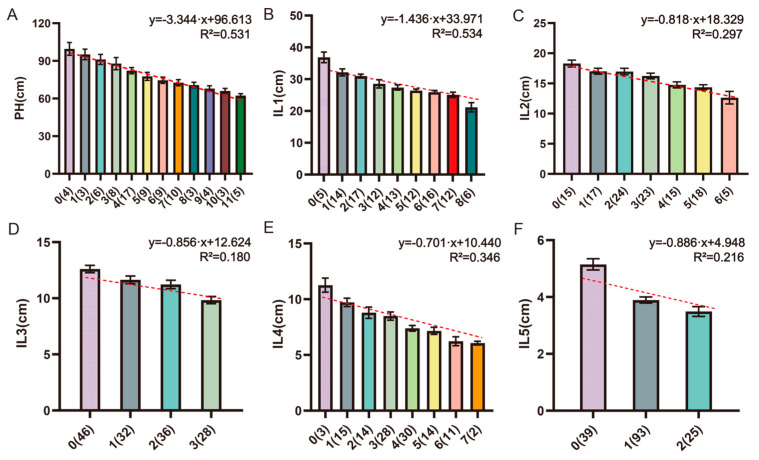
Aggregation effect analysis of stable QTL for plant height (PH, (**A**)) and five internode lengths (IL1–IL5, (**B**–**F**)).

To further elucidate the additive effects of the stable QTL, we analyzed and summarized the composition of favorable alleles (referring to reducing PH and IL) in RIL lines based on the closely linked marker genotypes of each identified locus. The favorable alleles of the 37 stable QTL were contributed by both BN4199 (22) and ZYM2 (15), which suggests that both parents provide different favorable alleles for PH and the five ILs (Table 2). Regardless of the interactions among these stable loci and environmental influences, we found that a greater number of favorable alleles was associated with a gradual decrease in PH and the five ILs (Figure 4), which supports the effectiveness of pyramid breeding. These results demonstrated that the total additive effects of the QTL combinations increased with the number of QTL. Additionally, most lines in the BN4199/ZYM2-RIL population carried four to seven favorable alleles for PH, while some individuals harbored up to 11 favorable alleles and could serve as valuable germplasm resources for wheat breeding.

### 3.4. Profiling and Identification of Candidate Genes

To identify putative candidate genes within the stable QTL intervals, the high-confidence annotated genes were screened based on the IWGSC CS RefSeq v2.1 genome assembly using the WheatOmics 1.0 platform. To further evaluate the functions of the annotated genes, we examined the cloning and functional analysis of homologous genes in rice. Furthermore, the QTG miner tool on the wGRN platform was used to prioritize high-confidence candidate genes, based on the previously reported genes associated with PH and IL in wheat. Additionally, the candidate gene was expressed in stem tissue during at least one distinct developmental stage. Collectively, a total of 63 high-confidence genes, including *Rht1*, *Rht8*, *TB1* and *ZnF-B*, were identified as potential candidates within the stable QTL regions in this study (Table 4).

### 3.5. Expression Pattern Analysis of Candidate Genes

**Figure 5:**
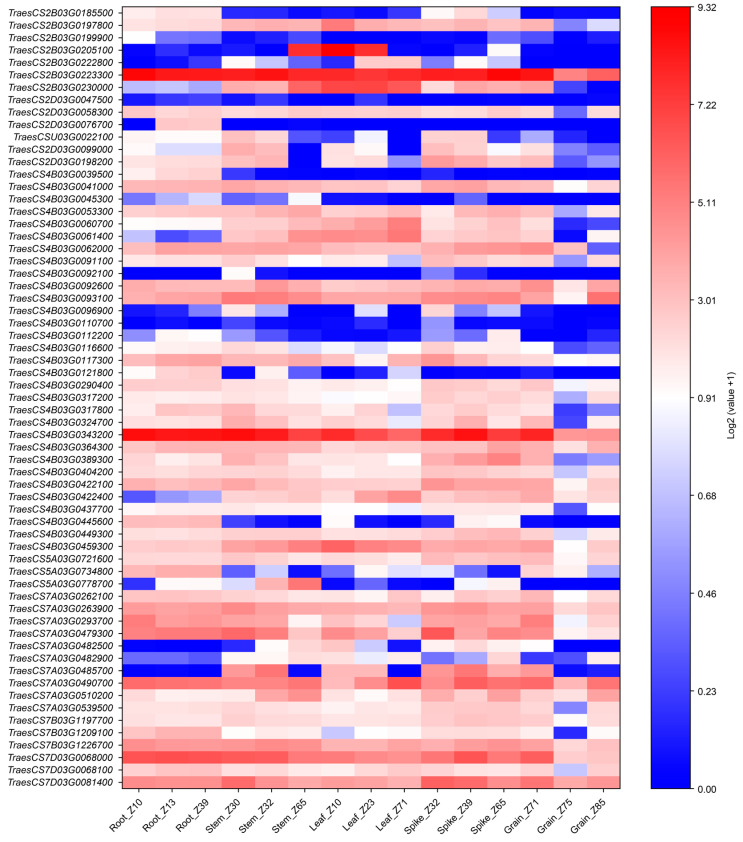
Heatmap of candidate gene expression across different tissues and developmental stages.

The expression profiles of these 63 candidate genes in different tissues and developmental stages were further analyzed using a publicly available CS wheat gene expression dataset from the WheatOmics 1.0 platform (Figure 5, Table S3). Among these, three genes (*TraesCS2B03G0223300*, *TraesCS4B03G0343200*, and *TraesCS7D03G0068000*) showed high expression in various tissues, whereas two genes (*TraesCS2D03G0047500* and *TraesCS4B03G0092100*) exhibited low expression across multiple tissues. Moreover, *TraesCS2B03G0205100* was mainly expressed in stem (Z65) and leaf (Z10 and Z23). As the stem develops, the expression levels of *TraesCS5A03G0778700*, *TraesCS7A03G0482500*, and *TraesCS7A03G0510200* increased markedly, while those of *TraesCS4B03G0324700*, *TraesCS4B03G0389300*, *TraesCS7A03G0479300*, and *TraesCS7D03G0081400* decreased obviously. Additionally, we selected the top ten candidate genes with high expression in the stems for RT-qPCR analysis (Table S4). The results showed that six of them (*TraesCS2B03G0223300*, *TraesCS7A03G0263900*, *TraesCS7A03G0479300*, *TraesCS7A03G0485700*, *TraesCS7D03G0068000*, and *TraesCS7D03G0081400*) exhibited significantly different expression levels between the two parents in the stem at the jointing stage (*p* < 0.05).

## 4. Discussion

### 4.1. QTL Cluster and Co-Localized QTL

As an important agronomic trait, PH plays a critical role in determining plant architecture and grain yield. Regardless of spike length, the final PH is biologically determined by the length of each internode. Moreover, plant architecture and lodging resistance in crops are greatly influenced by internode characteristics [58,59]. In this study, correlation analysis indicated that PH was significantly positively correlated with the length of each internode (Figure 2). Therefore, genetic dissection and identification of stable QTL and candidate genes for internode length will facilitate the improvement of the optimal plant height in wheat.

For closely related traits in crops, the co-localization of QTL or QTL clusters has been reported in various studies, which suggests potential pleiotropy or a tight linkage [60,61,62,63]. These regions, known as QTL hotspots, are critical for breeding and understanding the genetic architecture of main agronomic traits. In this study, a total of 23 co-localized QTL associated with at least two traits were identified on chromosomes 1B, 1D, 2D, 4A, 4B, 4D, 5A, 6B, 6D, 7A, and 7B (Table 3). Most co-localized QTL were associated with PH and specific ILs, particularly those of the two nodes below the spike. Furthermore, four co-localized QTL on chromosomes 2DS, 4BS, and 7BS simultaneously regulate PH and four ILs, which is consistent with the results of the correlation analysis between PH and ILs (Figure 2). Notably, seven co-localized QTL on chromosomes 1B, 2D, 4B, 5A, 6B, 6D, and 7A were associated only with internode length, and these loci were detected under specific environmental conditions (Table 3 and Table S2), which suggests that environmental factors significantly impact the expression of certain genes. In a previous study, conditional QTL mapping for plant height with respect to spike and internode length showed that spike length contributed the least to PH among the internode lengths considered at the QTL level [12]. In addition, the QTL cluster regions were found on chromosomes 2D, 4B, 5A, 7A, and 7B (Table 3, Figure 3), consistent with previous reports using different genetic populations [45,62,64]. These results indicate that these chromosomal regions may contain multiple key genes controlling PH and ILs, warranting further investigation.

### 4.2. QTL Comparison Analysis and Novel Loci Identification

As plant height in wheat is a complex quantitative trait controlled by multiple genes and QTL, many studies have been conducted to dissect its genetic basis [12,31,59]. In the present work, a total of 37 stable QTL associated with PH and ILs were identified, 26 of which were consistent with QTL regions previously reported (Table 2). For *QIl2.zzu.1B.2*, *QPh.zzu.5A.1*, *QIl4.zzu.5A.2*, *QIl4.zzu.7A.2*, and *QIl5.zzu.7A.5*, genomic loci associated with PH were also identified in a genome-wide association study using a panel of 287 wheat accessions collected over the past 100 years [55]. Furthermore, two QTL (*QPht/Sl.cau-2D.1* and *QPht/Sl.cau-2D.2*) linked in the coupling phase on chromosome 2DS with pleiotropic effects on PH and SL were separated and characterized using two near-isogenic line (NIL) pairs in a previous study [64]. Similarly, this study detected a QTL cluster on chromosome 2DS, which includes three co-localized stable QTL intervals (Table 3, Figure 3). Compared to previous findings, we speculate that this co-localized region (*QPh.zzu.2D.1* and *QIl1.zzu.2D.1*) may be a new genomic locus for PH and IL. In addition, 65 QTL-rich clusters (QRC) for PH were curated by thoroughly summarizing dwarfing loci from QTL linkage analyses and genome-wide association studies published from 2003 to 2022 [31]. By comparing with the QRC locations according to the IWGSC RefSeq v2.1, two co-localized stable QTL intervals on chromosomes 4BS (*QPh.zzu.4B.1*, *QIl2.zzu.4B.1*, *QIl3.zzu.4B.1* and *QIl4.zzu.4B.1*) and 7BL (*QPh.zzu.7B.1*, *QIl1.zzu.7B.1*, *QIl2.zzu.7B.1* and *QIl3.zzu.7B.1*) were identified as potentially novel loci in this study.

### 4.3. Promising Candidate Genes Associated with PH and ILs

With the rapid development of wheat genomics research, including genetic and physical mapping, whole-genome sequencing, and the advent of pan-omics technologies, comparative genomics research and gene discovery in wheat have been greatly facilitated [65,66]. In this work, based on gene function annotation, the cloning and identification of rice orthologous genes, analysis using the QTG miner module, and gene expression pattern analysis, a total of 63 potential candidate genes were prioritized within the stable QTL intervals (Table 4, Figure 5). For *QPh.zzu.2D.4* and *QPh.zzu.4B.3*, the ‘Green Revolution’ gene *Rht1* and the alternative gene *Rht8* were identified in the QTL regions of chromosomes 4BS and 2DS, respectively [21,29]. Furthermore, it was demonstrated that the *TB1* gene with the *QPh.zzu.4B.3* region regulates PH and stem internode length in bread wheat using pVRN1:TB-D1 transgenic lines [11]. The *ZnF-B* gene, which encodes a RING-type E3 ligase within the r-e-z haploblock, regulates PH via the BR signaling pathway [9]. Notably, the candidate gene *TraesCS4B03G0091100*, located near the *TB1* gene, encodes a phosphatidylinositol 4-phosphate 5-kinase 1. Its orthologous rice gene *Os03g0705300* (*OsPIP5K1*) has been reported to act with DWT1 and/or DWL2 to co-ordinately regulate the uniform growth of rice shoots through nuclear phosphoinositide signals [67]. Likewise, multiple candidate genes within the stable QTL intervals on chromosome 4B were listed (Table 4).

As one of the largest families of transcription factors in plants, the basic helical–loop–helical (bHLH) proteins play an important role in modulating BR signaling [68,69]. In addition to the *Rht8* gene, *TraesCS2D03G0198200*, a candidate gene within the *QPh.zzu.2D.4* interval, encodes the transcription factor bHLH148. A recent study showed that knockout mutants of the orthologous rice gene *Os03g0311600* (*OsAIF1*; *OsbHLH176*) had lower PH than the wild type but an elongated fifth internode [68]. Furthermore, the candidate gene *TraesCS7A03G0263900* within the *QPh.zzu.7A.1* interval encodes mitogen-activated protein kinase 1. Mitogen-activated protein kinase (MAPK) cascades are key signaling modules associated with numerous responses, including abiotic and biotic stress, hormonal changes, and developmental processes [70,71]. A *dwarf and small grain1* (*dsg1*) mutant in rice was identified and found to encode the mitogen-activated protein kinase OsMAPK6, which affects BR homeostasis and signaling [72]. For *QPh.zzu.5A.1*, a candidate gene that encodes a respiratory burst oxidase homolog protein C was identified. The orthologous rice gene *Os11g0537400* (*OsrbohI; Osrboh8*) was demonstrated to regulate rice growth via the jasmonic acid (JA) synthesis and signaling pathways [73]. Overall, further studies are needed to confirm the roles of the candidate genes identified in this study through functional approaches such as mutant screening, gene editing, and virus-induced gene silencing (VIGS).

## 5. Conclusions

In the present study, PH and its component ILs were evaluated in a set of 184 RILs derived from BN4199/ZYM2 across two field locations over two years, revealing significantly positive correlations among PH and the five ILs’ traits. The *H*^2^ of PH and the five ILs in the RIL population all exceeded 80%. A total of 37 stable QTL were identified on chromosomes 1B, 2B, 2D, 4B, 5A, 7A, 7B and 7D, accounting for 3.86–25.97% of the phenotypic variation. Meanwhile, 23 co-localized QTL associated with at least two traits were detected, and the QTL cluster regions were found on chromosomes 2D, 4B, 5A, 7A, and 7B. Additionally, based on gene function annotation, the cloning and identification of rice orthologous genes, and analysis using the QTG miner module, a total of 63 potential candidate genes (e.g., *Rht1*, *Rht8*, *TB1* and *ZnF-B*) were prioritized within the stable QTL intervals, and their tissue expression patterns were analyzed. These results deepen our understanding of the genetic basis of PH and ILs in wheat and provide a foundation for the further validation and functional characterization of candidate genes.

## Figures and Tables

**Table 1:** Heritability (*H*^2^) for plant height (PH) and five internode lengths (ILs) in the BN4199/ZYM2-RIL population.

Trait	Source	DF	SS	MS	F-Value	PCV	GCV	*H* ^2^
PH	Genotype	183	289,475.1875	1581.8317	54.1062 ***	16.95%	14.23%	96.57%
	Environment	3	25,489.6895	8496.5635	290.6229 ***			
	GE_interaction	530	29,199.0645	55.0926	1.8844 ***			
	Block/Env	8	5115.3491	639.4186	21.8712 ***			
	Error	1367	39,965.1953	29.2357				
IL1	Genotype	183	37,321.4844	203.9425	36.316 ***	20.70%	13.98%	92.82%
	Environment	3	16,399.9805	5466.6602	973.4479 ***			
	GE_interaction	530	8132.6426	15.3446	2.7324 ***			
	Block/Env	8	1014.4549	126.8069	22.5805 ***			
	Error	1367	7676.7583	5.6158				
IL2	Genotype	183	14,107.5928	77.0907	31.8138 ***	20.05%	14.77%	91.28%
	Environment	3	1555.4806	518.4935	213.9721 ***			
	GE_interaction	530	3781.9561	7.1358	2.9448 ***			
	Block/Env	8	154.5643	19.3205	7.9732 ***			
	Error	1367	3312.4912	2.4232				
IL3	Genotype	183	11,218.9746	61.3059	33.6283 ***	23.23%	18.82%	95.19%
	Environment	3	545.6771	181.8924	99.774 ***			
	GE_interaction	530	1593.6311	3.0069	1.6494 ***			
	Block/Env	8	144.9646	18.1206	9.9397 ***			
	Error	1367	2492.1011	1.823				
IL4	Genotype	183	7947.2256	43.4275	27.1927 ***	29.64%	22.54%	93.62%
	Environment	3	758.905	252.9683	158.3999 ***			
	GE_interaction	530	1510.5769	2.8501	1.7847 ***			
	Block/Env	8	155.5675	19.4459	12.1764 ***			
	Error	1367	2183.1313	1.597				
IL5	Genotype	183	2958.8389	16.1685	11.8842 ***	43.03%	26.28%	88.27%
	Environment	3	714.0789	238.0263	174.9542 ***			
	GE_interaction	530	1042.528	1.967	1.4458 ***			
	Block/Env	8	143.5233	17.9404	13.1866 ***			
	Error	1324	1801.3099	1.3605				

DF: Degree of freedom; SS: Sum of square; MS: Mean sum of square; PCV: Phenotypic coefficients of variation; GCV: Genotypic coefficients of variation. *H*^2^: Heritability. ***: Significant at the *p* < 0.001 level.

**Table 2:** Stable QTL for plant height (PH) and five internode lengths (ILs) in the BN4199/ZYM2-RIL population.

Trait	QTL Name	Chromosome	Genetic Interval (cM)	Physical Interval (Mb)	LOD	Additive Effect	R^2^	Environment	Reference
PH	*QPh.zzu.2D.1*	2D	1.3~7.8	9.7~12.9	4.60~7.39	3.28~4.71	7.93~12.95%	E1, E2, E3, E4, BLUE	
	*QPh.zzu.2D.2*	2D	10.8~16.2	14.5~16.7	5.10~6.51	3.70~4.14	9.47~10.91%	E2, E4, BLUE	[45]
	*QPh.zzu.2D.4*	2D	33.9~65.5	26.8~50.4	3.07~3.62	3.20~3.44	5.14~5.39%	E1, E2	[21,29]
	*QPh.zzu.4B.1*	4B	2.0~15.4	12.2~16.8	3.16~3.60	−3.12~−2.65	5.14~5.38%	E1, E3	
	*QPh.zzu.4B.2*	4B	16.3~24.1	16.8~22.4	2.62~3.14	−2.96~−2.46	4.61~4.62%	E1, E3	[46]
	*QPh.zzu.4B.3*	4B	30.7~48	26.0~54.0	2.90~4.40	−3.23~−2.62	4.64~7.13%	E2, E4, BLUE	[2]
	*QPh.zzu.5A.1*	5A	136.3~141.8	503.7~513.2	2.79~4.35	2.42~3.59	3.96~6.63%	E1, E2, BLUE	[32,47,48]
	*QPh.zzu.7A.1*	7A	99.2~116.7	68.0~86.5	2.58~2.87	−2.80~−2.48	4.16~4.31%	E2, E4	[49,50]
	*QPh.zzu.7A.2*	7A	116.7~128.8	86.5~99.9	2.57~2.68	−2.74~−2.45	3.86~4.02%	E2, E4	[51]
	*QPh.zzu.7B.1*	7B	121.8~135.7	714.0~717.1	3.53~5.61	−4.13~−2.90	5.59~9.66%	E1, E2, E4, BLUE	
	*QPh.zzu.7B.3*	7B	139.3~149.1	719.1~735.9	3.94~4.24	−3.62~−3.20	6.84~7.92%	E1, E4, BLUE	[19,52]
IL1	*QIl1.zzu.2B.1*	2B	67.4~78.7	48.2~69.9	3.60~5.49	0.94~1.40	5.83~8.72%	E3, E4	[53]
	*QIl1.zzu.2D.1*	2D	0.2~4.3	8.8~10.3	3.91~14.11	1.49~2.16	5.51~23.60%	E1, E2, BLUE	
	*QIl1.zzu.2D.2*	2D	10.9~14.2	14.5~16.5	5.55~13.24	1.67~2.65	10.76~25.97%	E1, E2, BLUE	[45]
	*QIl1.zzu.2D.3*	2D	18.7~23.5	16.5~19.9	3.51~5.05	0.98~1.73	5.91~7.18%	E1, E3	[45]
	*QIl1.zzu.2D.4*	2D	24.8~46.5	20.4~35.2	3.75~6.27	1.32~1.75	6.03~13.05%	E1, E3, E4	[21,29]
	*QIl1.zzu.7B.1*	7B	124.4~134.3	714.0~719.2	4.43~5.01	−1.37~−1.06	7.35~8.09%	E3, E4	
	*QIl1.zzu.7B.3*	7B	138.6~148.6	719.1~735.9	3.13~6.14	−1.64~−0.92	5.62~11.59%	E2, E3, E4	[19,52]
	*QIl1.zzu.7D.1*	7D	8.6~23.3	13.0~28	3.03~4.66	0.91~1.31	5.60~7.76%	E3, E4	[54]
IL2	*QIl2.zzu.1B.2*	1B	146.7~155.8	569.1~572.7	3.49	−0.80~−0.65	6.50~6.70%	E2, E4	[55]
	*QIl2.zzu.2D.2*	2D	8.6~16.2	13.4~16.5	2.53~3.33	0.57~0.73	5.18~5.30%	E1, E4	[45]
	*QIl2.zzu.4B.1*	4B	5.1~15.9	12.2~16.8	3.66~5.00	−0.87~−0.71	6.50~7.89%	E1, E2, BLUE	
	*QIl2.zzu.4B.2*	4B	17.9~23.9	17.4~22.7	2.73~4.25	−0.82~−0.64	5.03~6.89%	E1, E2, BLUE	[46]
	*QIl2.zzu.4B.3*	4B	64.1~68.8	162.1~310.5	3.65~4.83	−0.94~−0.64	6.12~9.27%	E3, E4, BLUE	[56,57]
	*QIl2.zzu.7B.1*	7B	120.8~135.7	714.0~718.7	2.79~6.38	−1.00~−0.69	5.27~11.33%	E1, E3, BLUE	
IL3	*QIl3.zzu.4B.1*	4B	4.7~15.7	12.2~16.8	3.38~7.62	−1.02~−0.59	6.14~14.17%	E1, E2, E3, E4, BLUE	
	*QIl3.zzu.4B.2*	4B	18.1~32.5	17.7~28.0	4.17~6.94	−1.04~−0.67	7.96~12.72%	E1, E2, E3, E4, BLUE	[46]
	*QIl3.zzu.7B.1*	7B	124.8~135.7	714.0~718.7	2.98~6.32	−0.76~−0.52	5.10~10.77%	E1, E4, BLUE	
IL4	*QIl4.zzu.4B.1*	4B	7.6~16.3	12.2~16.8	3.20~4.72	−0.70~−0.51	5.65~8.00%	E1, E3	
	*QIl4.zzu.4B.2*	4B	19.2~23.9	17.7~22.4	3.37~3.74	−0.65~−0.52	5.97~6.86%	E1, E3	[46]
	*QIl4.zzu.4B.3*	4B	63.9~68.4	144.0~357.5	3.10~4.12	−0.55~−0.46	5.16~7.15%	E2, E4	[56,57]
	*QIl4.zzu.5A.2*	5A	136.6~141.8	503.7~513.2	3.92~5.33	0.57~0.77	6.92~9.09%	E1, E3	[32,47,48]
	*QIl4.zzu.5A.3*	5A	145.4~148.9	533.0~535.8	3.64~4.05	0.55~0.67	6.40~7.01%	E1, E3	
	*QIl4.zzu.7A.1*	7A	97.8~107.4	68.0~82.7	5.14~7.31	−0.73~−0.63	9.24~13.09%	E2, E4	[49,50]
	*QIl4.zzu.7A.2*	7A	159.8~171.9	180.6~206.7	2.53~5.15	0.41~0.63	4.31~9.17%	E2, E4	[55]
IL5	*QIl5.zzu.7A.3*	7A	99.2~107.9	78.5~82.7	6.23~11.90	−0.55~−0.45	11.09~20.33%	E1, E2, E4, BLUE	[49,50]
	*QIl5.zzu.7A.6*	7A	160.3~169.3	168.7~218.5	3.30~6.25	0.32~0.44	7.14~11.11%	E2, E4, BLUE	[55]

Note: A positive additive effect indicates that the increasing allele of the corresponding QTL is contributed by Zhengyinmai 2 (ZYM2), whereas a negative additive effect indicates that the increasing allele is contributed by Bainong 4199 (BN4199).

**Table 3:** Summary of co-localized QTL regions identified for plant height (PH) and five internode lengths (ILs).

Chromosome	Trait	Genetic Interval (cM)	Physical Interval (Mb)	QTL Name
1B	IL3, IL4	159.9~170.8	578.4~592.8	*QIl3.zzu.1B.1*; *QIl4.zzu.1B.1*
1D	PH, IL1, IL3	145.4~154.6	401.3~414.1	*QPh.zzu.1D*; *QIl1.zzu.1D.2*; *QIl3.zzu.1D*
2D	PH, IL1, IL2, IL4, IL5	0~7.8	8.8~12.9	*QPh.zzu.2D.1*; *QIl1.zzu.2D.1*; *QIl2.zzu.2D.1*; *QIl4.zzu.2D.1*; *QIl5.zzu.2D.1*
2D	PH, IL1, IL2, IL5	8.5~16.2	13.4~16.5	*QPh.zzu.2D.2*; *QIl1.zzu.2D.2*; *QIl2.zzu.2D.2*; *QIl5.zzu.2D.2*
2D	PH, IL1, IL5	18.7–29.5	16.5~26.8	*QPh.zzu.2D.3; QIl1.zzu.2D.3; QIl5.zzu.2D.3*
2D	PH, IL1, IL4, IL5	23.5–65.5	26.8~50.4	*QPh.zzu.2D.4; QIl1.zzu.2D.4; QIl4.zzu.2D.2; QIl5.zzu.2D.4*
2D	IL4, IL5	78.1~91.9	58.5~67.7	*QIl4.zzu.2D.3; QIl5.zzu.2D.5*
4A	PH, IL2, IL3, IL4	85.5~98.6	598.7~610.9	*QPh.zzu.4A.1; QIl2.zzu.4A; QIl3.zzu.4A; QIl4.zzu.4A*
4B	PH, IL1, IL2, IL3, IL4	2~16.3	12.2~16.8	*QPh.zzu.4B.1; QIl1.zzu.4B.1; QIl2.zzu.4B.1; QIl3.zzu.4B.1; QIl4.zzu.4B.1*
4B	PH, IL1, IL2, IL3, IL4	16.3~32.5	16.8~28	*QPh.zzu.4B.2; QIl1.zzu.4B.2; QIl2.zzu.4B.2; QIl3.zzu.4B.2; QIl4.zzu.4B.2*
4B	PH, IL3	30.3~48	28.0~54.0	*QPh.zzu.4B.3; QIl3.zzu.4B.3*
4B	IL1, IL2, IL3, IL4	63.1~68.9	144.0~357.5	*QIl1.zzu.4B.3; QIl2.zzu.4B.3; QIl3.zzu.4B.4; QIl4.zzu.4B.3*
4D	PH, IL3, IL4	0~1.4	37.3~42.5	*QPh.zzu.4D.1; QIl3.zzu.4D.1; QIl4.zzu.4D*
5A	PH, IL3, IL4	136.3~141.8	503.7~513.2	*QPh.zzu.5A.1; QIl3.zzu.5A.1; QIl4.zzu.5A.2*
5A	IL4, IL5	145.4~148.9	525.6~535.8	*QIl4.zzu.5A.3; QIl5.zzu.5A.2*
5A	PH, IL2, IL3	151.2~157	539.6~550.0	*QPh.zzu.5A.3; QIl2.zzu.5A; QIl3.zzu.5A.2*
6B	IL1, IL3, IL4	195.7~206.6	703.6~714.7	*QIl1.zzu.6B.3; QIl3.zzu.6B; QIl4.zzu.6B*
6D	IL2, IL3, IL4	156.1~172.3	454.6~469.8	*QIl2.zzu.6D; QIl3.zzu.6D; QIl4.zzu.6D*
7A	PH, IL4, IL5	96.2~116.7	68.0~86.5	*QPh.zzu.7A.1; QIl4.zzu.7A.1; QIl5.zzu.7A.2*
7A	PH, IL1, IL3, IL4, IL5	159.8~176.8	143.2~237.1	*QPh.zzu.7A.3; QIl1.zzu.7A.1; QIl3.zzu.7A; QIl4.zzu.7A.2; QIl5.zzu.7A.6*
7A	PH, IL4	203.3~210.5	613.2~639.1	*QPh.zzu.7A.6; QIl4.zzu.7A.4*
7B	PH, IL1, IL2, IL3, IL4	120.7~135.7	714.8~717.7	*QPh.zzu.7B.1; QIl1.zzu.7B.1; QIl2.zzu.7B.1; QIl3.zzu.7B.1; QIl4.zzu.7B*
7B	PH, IL1, IL2, IL3	139.3~149.1	719.1~735.9	*QPh.zzu.7B.3; QIl1.zzu.7B.3; QIl2.zzu.7B.3; QIl3.zzu.7B.2*

**Table 4:** The putative candidate genes identified within the stable QTL regions.

Gene ID	Gene Name	Location (IWGSC RefSeqv2.1)	Description	Oryza Sativa
*TraesCS2B03G0185500*		chr2B:51952971-51956065 (−)	Transcription factor MYB36	*Os08g0433400*
*TraesCS2B03G0197800*		chr2B:56466313-56470936 (−)	GDT1-like protein 2, chloroplastic	*Os11g0544500*
*TraesCS2B03G0199900*		chr2B:57026094-57027547 (+)	UDP-glycosyltransferase 79	*Os04g0206700*
*TraesCS2B03G0205100*		chr2B:58334563-58335201 (+)	Germin-like protein 8–14	*Os08g0460000*
*TraesCS2B03G0222800*		chr2B:63367187-63468933 (−)	Two-component response regulator-like PRR37	*Os07g0695100*
*TraesCS2B03G0223300*		chr2B:63771237-63775184 (+)	L-ascorbate peroxidase 2, cytosolic	*Os07g0694700*
*TraesCS2B03G0230000*		chr2B:66373336-66376648 (−)	Omega-3 fatty acid desaturase, chloroplastic	*Os07g0693800*
*TraesCS2D03G0047500*		chr2D:11057729-11060001 (−)	Probably inactive receptor-like protein kinase At2g46850	*Os10g0351500*
*TraesCS2D03G0058300*		chr2D:12785126-12799210 (−)	ABC transporter B family member 25, mitochondrial	*Os06g0128300*
*TraesCS2D03G0076700*		chr2D:15339456-15341024 (+)	S-adenosylmethionine synthase 1	*Os01g0323600*
*TraesCSU03G0022100*	*Rht8*	chrUn:18733736-18737027 (−)	Ribonuclease H-Like 1	*Os04g0261400*
*TraesCS2D03G0099000*		chr2D:20858057-20860133 (+)	Tricetin 3′,4′,5′-O-trimethyltransferase	*Os08g0157500*
*TraesCS2D03G0198200*		chr2D:49296329-49297483 (+)	Transcription factor bHLH148	*Os03g0311600*
*TraesCS4B03G0039500*		chr4B:15737018-15740036 (−)	Fe(2+) transport protein 1	*Os03g0667500*
*TraesCS4B03G0041000*		chr4B:15848203-15853551 (−)	Guanine nucleotide-binding protein subunit beta	*Os03g0669200*
*TraesCS4B03G0045300*		chr4B:17485639-17488430 (+)	Endo-1,4-beta-xylanase 1	*Os03g0672900*
*TraesCS4B03G0053300*		chr4B:19889929-19895453 (−)	BEL1-like homeodomain protein 6	*Os03g0680800*
*TraesCS4B03G0060700*		chr4B:23432237-23437771 (+)	Magnesium transporter MRS2-A, chloroplastic	*Os03g0684400*
*TraesCS4B03G0061400*		chr4B:23847772-23850308 (+)	Ferredoxin C 2, chloroplastic	*Os03g0685000*
*TraesCS4B03G0062000*		chr4B:23945006-23948825 (+)	Gamma-glutamyl peptidase 5	*Os03g0685300*
*TraesCS4B03G0091100*		chr4B:32026724-32033370 (−)	Phosphatidylinositol 4-phosphate 5-kinase 1	*Os03g0705300*
*TraesCS4B03G0092100*	*TB1*	chr4B:33121434-33122498 (+)	Transcription factor TB1	*Os03g0706500*
*TraesCS4B03G0092600*	*ZnF-B*	chr4B:33478324-33487967 (−)	RING-type E3 ligase	*Os03g0706900*
*TraesCS4B03G0093100*	*Rht1*	chr4B:33614435-33616890 (+)	DELLA protein RHT-1	*Os03g0707600*
*TraesCS4B03G0096900*		chr4B:34988758-34989243 (−)	Chemocyanin	*Os03g0709100*
*TraesCS4B03G0110700*		chr4B:42801164-42805060 (−)	ATP-dependent DNA helicase DDM1	*Os03g0722400*
*TraesCS4B03G0112200*		chr4B:43560903-43567074 (−)	Phytochrome A type 3	*Os03g0719800*
*TraesCS4B03G0116600*		chr4B:45598303-45600496 (−)	SCARECROW-LIKE protein 7	*Os03g0723000*
*TraesCS4B03G0117300*		chr4B:45930921-45934993 (−)	Uncharacterized sugar kinase slr0537	*Os01g0105900*
*TraesCS4B03G0121800*		chr4B:48378594-48380754 (+)	7-methyl-GTP pyrophosphatase	*Os03g0724700*
*TraesCS4B03G0290400*		chr4B:155152087-155157174 (−)	OBERON-like protein	*Os12g0514400*
*TraesCS4B03G0317200*		chr4B:181393820-181403278 (−)	E3 SUMO-protein ligase SIZ2	*Os03g0719100*
*TraesCS4B03G0317800*		chr4B:182163392-182169863 (+)	Protein DWARF 53	*Os11g0104300*
*TraesCS4B03G0324700*		chr4B:186822081-186825362 (−)	GDSL esterase/lipase CPRD49	*Os11g0708400*
*TraesCS4B03G0343200*		chr4B:202475060-202477534 (+)	UDP-arabinopyranose mutase 1	*Os03g0599800*
*TraesCS4B03G0364300*		chr4B:224505364-224506622 (−)	Protein ELF4-LIKE 4	*Os11g0621500*
*TraesCS4B03G0389300*		chr4B:254960827-254974470 (−)	Villin-2	*Os03g0356700*
*TraesCS4B03G0404200*		chr4B:277078935-277090963 (−)	Ubiquitin-like-specific protease ESD4	*Os03g0344300*
*TraesCS4B03G0422100*		chr4B:306200080-306210987 (+)	Histone chaperone domain CHZ	*Os11g0544600*
*TraesCS4B03G0422400*		chr4B:306823340-306826867 (−)	Inactive purple acid phosphatase-like protein	*Os11g0586300*
*TraesCS4B03G0437700*		chr4B:319887960-319893701 (+)	RNA polymerase II C-terminal domain phosphatase-like 3	*Os11g0521900*
*TraesCS4B03G0445600*		chr4B:327792210-327794025 (+)	Probable serine/threonine-protein kinase PBL15	*Os03g0364400*
*TraesCS4B03G0449300*		chr4B:335351963-335359341 (−)	Phosphatidate phosphatase PAH2	*Os11g0615000*
*TraesCS4B03G0459300*		chr4B:346726523-346748266 (−)	ATP-dependent Clp protease proteolytic subunit 6, chloroplastic	*Os03g0411500*
*TraesCS5A03G0721600*		chr5A:505345139-505349040 (+)	Hsp70 nucleotide exchange factor fes1	*Os09g0512700*
*TraesCS5A03G0734800*		chr5A:511126101-511128863 (−)	Respiratory burst oxidase homolog protein C	*Os11g0537400*
*TraesCS5A03G0778700*		chr5A:534224114-534227378 (−)	Protein ODORANT1	*Os09g0532900*
*TraesCS7A03G0262100*		chr7A:70990291-70993190 (−)	F-box/LRR-repeat MAX2 homolog	*Os06g0154200*
*TraesCS7A03G0263900*		chr7A:71403697-71411075 (−)	Mitogen-activated protein kinase 1	*Os06g0154500*
*TraesCS7A03G0293700*		chr7A:83704628-83709372 (+)	Protein disulfide isomerase-like 1-5	*Os06g0163400*
*TraesCS7A03G0479300*		chr7A:172295262-172296871 (−)	Ribonucleoside-diphosphate reductase small chain	*Os06g0257450*
*TraesCS7A03G0482500*		chr7A:174101472-174102770 (+)	Zinc finger protein CONSTANS-LIKE 16	*Os06g0264200*
*TraesCS7A03G0482900*		chr7A:174308926-174314065 (+)	Protein NRT1/ PTR FAMILY 3.1	*Os06g0264500*
*TraesCS7A03G0485700*		chr7A:175963414-175964402 (−)	Gibberellin-regulated protein 4	*Os06g0266800*
*TraesCS7A03G0490700*		chr7A:178822117-178827345 (+)	Aldehyde dehydrogenase family 2 member B4, mitochondrial	*Os06g0270900*
*TraesCS7A03G0510200*		chr7A:191343804-191349225 (−)	Zinc finger protein CONSTANS-LIKE 9	*Os06g0298200*
*TraesCS7A03G0539500*		chr7A:209112211-209132047 (+)	Ubiquitin-like-specific protease 1D	*Os06g0487900*
*TraesCS7B03G1197700*		chr7B:717798428-717806399 (−)	Zinc finger CCCH domain-containing protein 7	*Os06g0638000*
*TraesCS7B03G1209100*		chr7B:720834549-720838034 (−)	Receptor-like cytoplasmic kinase 176	*Os05g0110900*
*TraesCS7B03G1226700*		chr7B:725133900-725140584 (+)	Protein MEMO1	*Os08g0299000*
*TraesCS7D03G0068000*		chr7D:16298182-16301159 (−)	6-phosphogluconate dehydrogenase, decarboxylating 1	*Os06g0111500*
*TraesCS7D03G0068100*		chr7D:16346353-16351684 (+)	Extra-large guanine nucleotide-binding protein 3	*Os06g0111400*
*TraesCS7D03G0081400*		chr7D:19316635-19322955 (−)	Protein argonaute 1B	*Os04g0566500*

## Data Availability

The data are contained within the article and the supplementary materials.

## Supplementary Materials

The following supporting information can be downloaded at: https://www.mdpi.com/article/10.3390/genes17050567/s1, Table S1: Statistical analysis of plant height (PH) and five internode lengths (ILs) for parents and the RIL population across different environments; Table S2: QTL for plant height (PH) and five internode lengths (ILs) identified in the BN4199/ZYM2-RIL population across different environments; Table S3: Expression of candidate genes in various tissues; Table S4: List of primers for reverse transcription–quantitative polymerase chain reaction (RT-qPCR) analysis. Figure S1: RT-qPCR analysis of ten candidate genes in the stems of the parents ZYM2 and BN4199 during the jointing stage. *, and **: significant differences at the *p* < 0.05, and *p* < 0.01 levels, respectively. ns: not significant.

## Abbreviations

The following abbreviations are used in this manuscript:

PH	Plant height
IL	Internode length
SL	Spike length
RIL	Recombinant inbred line
QTL	Quantitative trait loci
IWGSC	International Wheat Genome Sequencing Consortium
CS	Chinese Spring
MAS	Marker-assisted selection
