# Supplementary figures and images for: Identification of QTL and Candidate Genes Controlling Plant Height and Internode Length in a Newly Characterized Bread Wheat Recombinant Inbred Population
Source: Genes (Basel). 2026 May 17;17(5):567. doi: 10.3390/genes17050567 (PMC13205224; doi:10.3390/genes17050567)

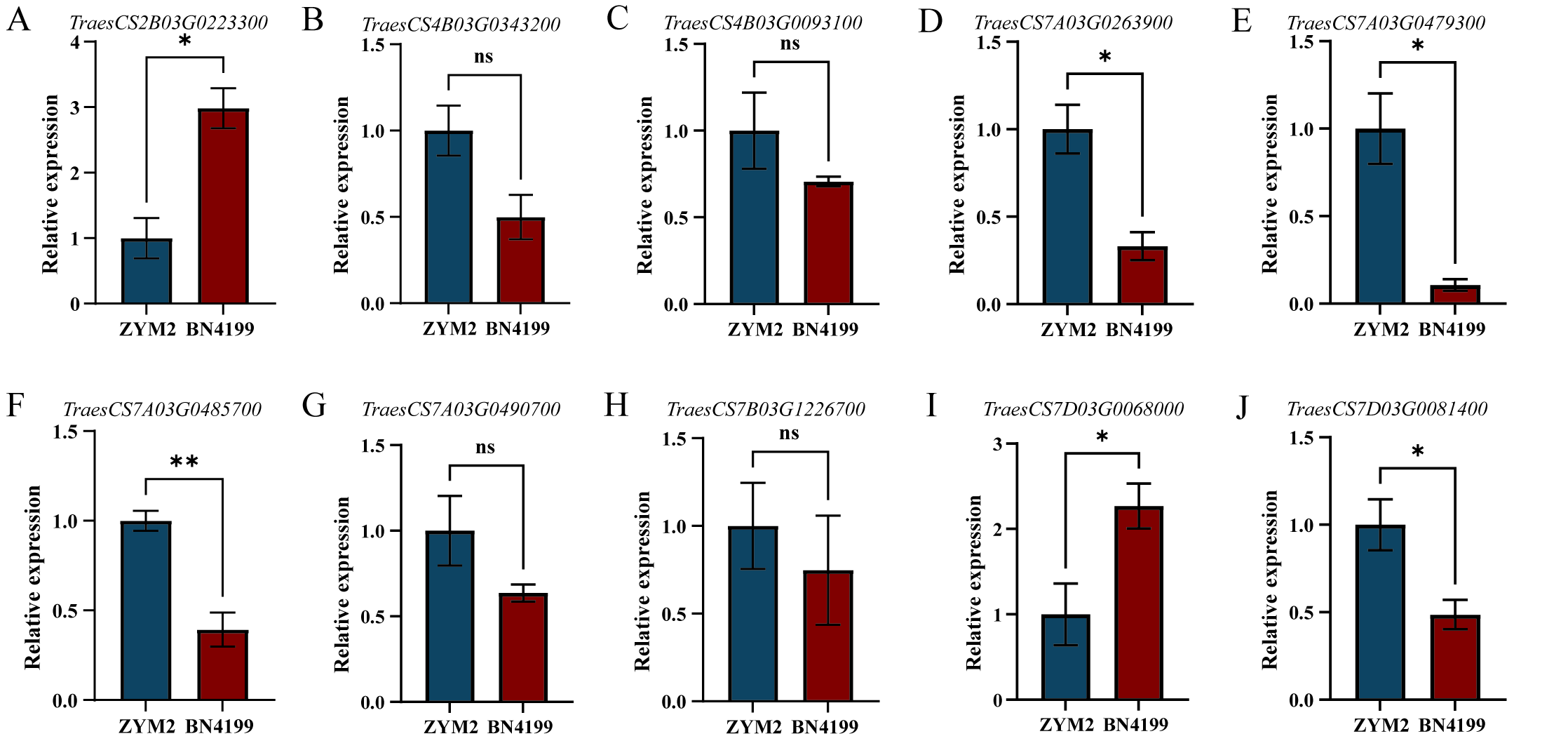

Supplement: Supplementary file 1 [file genes-17-00567-s001.zip › Supplementary Figure S1.tif]
